# Diagnostic performance of four AI tools in pharmacology MCQs: Accuracy, sensitivity, and specificity

**DOI:** 10.1371/journal.pone.0337688

**Published:** 2025-12-16

**Authors:** Ayah J. Al-Rahahleh, Mai Z. Rizik, Fahmi Y. Al-Ashwal, Rana K. Abu-Farha

**Affiliations:** 1 Clinical Pharmacy and Therapeutics Department, Faculty of Pharmacy, Applied Science Private University, Amman, Jordan; 2 Faculty of Education, University of Ottawa, Ottawa, Ontario, Canada; 3 Department of Clinical Pharmacy and Pharmacy Practice, University of Science and Technology, Sana’a, Yemen; 4 Department of Clinical Pharmacy, College of Pharmacy, Al-Ayen Iraqi University, Thi-Qar, Iraq; Northern Border University, SAUDI ARABIA

## Abstract

**Background:**

The rapid rise of AI in medical and pharmaceutical education has engendered much interest; however, a knowledge gap still exists in the evaluation of performances of these tools in critical academic contexts.

**Objectives:**

The aim of this study was to assess and compare the performances of four openly accessible AI language tools, Microsoft Copilot, ChatGPT-3.5, Google Gemini, and DeepSeek AI, in responding to pharmacology-related MCQs with regard to diagnostic accuracy, sensitivity, specificity, and reproducibility.

**Methods:**

A total of 80 MCQs were generated and validated, representing four therapeutic systems: cardiovascular, respiratory, gastrointestinal, and endocrine, including four pharmacological domains: mechanism of action, side effects, pharmacokinetics, and drug-drug interactions. Answers were classified into true/false positives and negatives in order to calculate accuracy, sensitivity, and specificity. After two weeks, a second round of testing was performed with the questions to assess answer reproducibility.

**Results:**

The top overall performer was Microsoft Copilot: 87.5% accuracy, a sensitivity of 94.6%, and a specificity of 70.8%. It continued to perform strongly across all therapeutic systems, especially in the cardiovascular and respiratory domains, with the highest accuracy in identifying drug mechanisms and side effects. ChatGPT-3.5 performed similarly to Google Gemini (76.3% and 75.0% accuracy, respectively) but with higher sensitivity for ChatGPT-3.5 and higher specificity for Gemini. DeepSeek AI had the lowest accuracy overall (68.8%) and the lowest specificity (29.2%), but the highest consistency of reproducibility (97.5%). The performance of all tools decreased significantly with increasing level of question difficulty (p < 0.05).

**Conclusion:**

All tools have some value in pharmacology education, but Microsoft Copilot was the most consistently accurate. Limitations in complexity and reproducibility suggest that caution should be exercised in academic and clinical use, particularly given the variability seen with ChatGPT-3.5.

## Introduction

Essentially, pharmacology refers to the study of the effects of drugs and other chemicals on living systems, especially on cell, tissue, and organ action [1]. In practice, pharmacology primarily serves to guide the clinician and the patient in safe and appropriate therapeutic approaches. It encompasses everything from identification and manufacturing of medications to selection, compounding, and dispensing of medications [2].

Rapid advances over the past couple years in the field of artificial intelligence (AI) have started to change parts of healthcare, including pharmacology. AI is generally defined as creating systems with the ability to imitate human cognitive functions such as learning, reasoning, and problem-solving [3] and is finding its way into healthcare-related fields from diagnostics to clinical decision support. One noteworthy milestone was the launch of ChatGPT, a large language model developed by OpenAI, in late 2022, which opened new doors of opportunity for AI in the medical field, education, and beyond [4].

The various areas where AI has found its application include diagnosis and development of new drugs, patient care, and administrative-related support activities in medicine, among others [5]. The emerging tools like ChatGPT, Microsoft Copilot, and Google Gemini are being explored for a range of applications in pharmacology, including selection of drugs, interaction checks, and answering of complex pharmacological questions [6,7]. Applications are particularly promising in education and clinical decision-making.

As these generative tools continue to improve, so does the functionality within the tools. To date, language tools like ChatGPT and multimodal tools like DALL·E are able to provide human-like responses, images, and even videos. Applications of the same include medical education, communication, and personalized treatment planning [8,9]. With enhanced Natural Language Processing (NLP), these tools are now capable of easily understanding complex queries and producing clinically relevant responses.

Given that AI tools are finding their way increasingly into medical practice, it is relevant to assess the performance of these tools in a high-stakes context, particularly for questions on pharmacology. AI-powered tools, such as ChatGPT-3.5, Microsoft Copilot, Google Gemini, and DeepSeek, are being leveraged to address various issues, ranging from side effects and mechanisms of action to pharmacokinetics and drug interactions. While there is a growing belief that these tools can perform at least as well as, or even outperform, human experts in specific areas of expertise [5], their actual effectiveness for specific pharmacology tasks has not been systematically evaluated yet. The purpose of this work has been to compare the sensitivity, specificity, and accuracy of four high-end AI tools in answering MCQs on pharmacology, primarily focusing on mechanisms of drug action, side effects, pharmacokinetics, and drug-drug interactions.

Prior research has looked into the performance of AI tools in cases of clinical pharmacy. In one case, Albogami et al., 2025, reported on the performance of GPT-3, GPT-3.5, and GPT-4 compared to licensed pharmacists in a case of clinical pharmacy, discovering that GPT-4 performed comparably with pharmacists, while the other versions did less well [10]. In a study on the Taiwan National Pharmacist Licensing Examination, GPT-4 was accurate 72.9% of the time compared to 86.7% of accurately awarded marks for GPT-3.5 in basic pharmacology, with the differences in clinical questions being small [11]. In contrast, a study at Chiang Mai University reported that the % (44%) of correct answers received by ChatGPT on a 4th-year pharmacy exam, in comparison to the percentage (66%) of correct answers received by students, indicated that even ChatGPT has weaknesses in some real-world situations [12]. This study makes several new contributions: it compares four AI tools side by side; uses validated MCQs sourced from authoritative pharmacological sources; uses newly measured metrics for sensitivity and specificity; and includes reproducibility metrics that assess both accuracy and consistency over time.

## Methods

### Study design and development of pharmacology questions

The present study comparatively assessed the performances of four of the newest AI tools in answering MCQs related to pharmacology. The research took place throughout April 2025. The principle investigator developed 80 MCQs on pharmacology (S1 Appendix). These items were developed from trusted and authoritative pharmacological sources, including UpToDate, Drugs.com, Lexicomp, and renowned textbooks such as Goodman & Gilman’s: The Pharmacological Basis of Therapeutics, Basic and Clinical Pharmacology by Bertram Katzung. All questions were written in English, which is the standard language of instruction in pharmacy programs across Jordan.

Every MCQ had four different options: A, B, C, and D, which may or may not include the correct answer. While all the questions did not explicitly state “None” as one of the multiple-choice options presented, the AI tools were not limited to choosing from only those provided. If the AI decided that none of the given options were correct, it was at liberty to write “None” or say that no option was correct.

The content of the questions spanned four major therapeutic systems: cardiovascular, respiratory, gastrointestinal, and endocrine systems. In addition to these clinical systems, each question also fell into one of four pharmacological categories: mechanism of action, side effects, pharmacokinetics, or drug-drug interactions.

To verify the validity of the multiple-choice questions (MCQs) as well as the veracity of the answers, both the MCQs and answers were independently evaluated by two full professors in pharmacology. They reviewed the MCQs based on their considerable experience in clinical pharmacy and consulted references. They both independently confirmed that all answers were correct. Since there was agreement, they provided verification of the correctness of all answers, the purpose of the review was limited to suggestions on improving clarity of the questions. Therefore, interrater agreement was not formally assessed across raters as the review did not involve assessments of discrepancies across answers.

### Pilot testing

The final set of MCQs was shared with five clinical pharmacy practitioners (Pharm D) ahead of the main evaluation to look at their perception about the clarity and structure of the questions. These practitioners also attempted the questions themselves. Analysis of the response from each was done to find out the difficulty level of each question through the formula for DI: DI = Number of correct responses/ Total number of responses. Later, questions were grouped into a series of scores of DI as: DI > 0.8 “Easy”, 0.4 < DI ≤ 0.8 “Moderate”, and DI ≤ 0.4 “Difficult.” This ensured that the final list of questions used in the evaluation of AI tools had a balanced spread of the questions in terms of their difficulty.

### AI tool selection, interaction, and testing

The free versions of four of the latest AI tools were used in this study: ChatGPT-3.5, Microsoft Copilot, Google Gemini, and DeepSeek AI. To guarantee consistency in interaction across the different tools, a standard prompt was designed and provided at the beginning of each conversation with each AI tool: “For the following pharmacology MCQ, please select the most appropriate answer. Note that there is only one correct answer. If you feel that none of the options is correct, please respond with “None.”

Each MCQ was entered into the AI tools separately using ‘New Chat’ feature to avoid memory retention from the previously asked questions. Upon receiving an answer, a screenshot was taken to document the response given by AI. In this way, a total of 320 screenshots were obtained. The responses generated by AI were categorized in the following classes: True Positive (TP): when the AI tool chooses the right answer from the four options available for which a correct response is available. False Negative (FN): when the AI selects an incorrect choice (either an incorrect option from the four options, or ‘None’), despite a correct response being available. True Negative (TN): when the AI correctly selects ‘None’ when no answer was deemed correct. False Positive (FP): when the AI selects a wrong answer from the options available, where no answer was correct.

The performance of each AI tool was computed based on accuracy, sensitivity, and specificity, as follows: Accuracy: Proportion of questions answered correctly by the AI tool [Accuracy = TP + TN/ (TP + TN + FP + FN)]. Sensitivity: The extent to which the AI tool can identify true positives, that is, the correct answers from all actual positives, that is, when a correct answer is available for a question [Sensitivity = TP/ (TP + FN)]. Specificity: The extent to which the AI tool can identify true negatives, that is, selection of “None” where no answer was correct out of all actual negatives where no answer was correct [Specificity = TN/ (TN + FP)].

Besides this, Cohen’s Kappa (κ) statistic was computed to measure agreement in the response of AI for each therapeutic area and all domains. Also, for reproducibility assessment, we re-tested the AI tools for the second time after two weeks from the first assessment. Each AI tool was presented with the same set of 80 MCQs using the identical prompt as before. The answers from both rounds were compared. Metrics for reproducibility assessment included the following: Reproducibility Index which is the proportion of identical answers of a particular AI tool between the two rounds. Since the questions are multiple-choice, an “identical answer” in this context means that the AI tool selects the same answer (A, B, C, D, or “None”) across both rounds of testing. Also, Intra-Rater Agreement using Cohen’s Kappa (κ) was calculated to find the level of agreement between the AI responses in both rounds.

### Statistical analysis

The responses collected from the AI tools were analyzed using IBM SPSS Statistics Version 22.0, from IBM Corp., Armonk, NY, USA. From a statistical point of view, the current study focused on four key points regarding the performance assessment: accuracy, sensitivity, specificity, and reproducibility index. Chi-square was used to identify the distribution of correct and incorrect responses for each AI tool throughout each level of difficulty. Intra-tool reproducibility was evaluated using Cohen’s Kappa, which judged the consistency of each AI tool’s answers between the first and second rounds of testing with the same set of 80 MCQs. The McNemar test was then conducted to identify possible variations in tool accuracy between the two test runs. For all tests, p ≤ 0.05 was considered statistically significant.

## Results

In the pilot testing, a total of 80 MCQs were analyzed for their DI. Out of these, 27 questions (33.8%) were categorized as easy (DI > 0.8), 47 questions (58.8%) as average (0.4 < DI ≤ 0.8), and 6 questions (7.5%) as difficult (DI ≤ 0.4). In each therapeutic system, the cardiovascular system consisted of 8 easy, 10 average, and 2 difficult questions, while the gastrointestinal system had 7 easy, 10 average, and 3 difficult questions. The respiratory system had 5 easy, 14 average, and 1 difficult question. Finally, the endocrine system had 7 easy and 13 average questions, with no difficult items.

**Table 1** presents the confusion matrix for the four AI tools in answering pharmacology questions. Microsoft Copilot had the highest correct predictions, with 53 TP and 17 TN, followed by ChatGPT-3.5, which had 52 TP and 7 TN, Google Gemini with 49 TP and 10 TN, and DeepSeek AI with 48 TP and 7 TN.

**Table 1:** AI tool confusion matrix for predicting correct and “none” answers.

AI Tool	True Positives (TP)	False Negatives (FN)	True Negatives (TN)	False Positives (FP)
ChatGPT-3.5	52	4	7	17
Google Gemini	49	7	10	14
Microsoft Copilot	53	3	17	7
DeepSeek AI	48	8	7	17

The performance of the AI tools is summarized in **Table 2**, including metrics on accuracy, sensitivity, and specificity. Microsoft Copilot had the highest rate on all three metrics: accuracy was 87.5%, sensitivity was 94.6%, and specificity was 70.8%. DeepSeek AI had the lowest values for accuracy (68.8%) and specificity (29.2%), with a sensitivity of 85.7%. Both ChatGPT-3.5 and Google Gemini correctly identified 73.8% of the questions, but ChatGPT-3.5 had higher sensitivity, 92.9%, and lower specificity, 29.2%, than Google Gemini, with a specificity of 41.7%.

**Table 2:** AI tool performance summary (accuracy, sensitivity, and specificity).

AI Tool	Metric	Value	95% CI
ChatGPT-3.5	Accuracy	73.8%	[64.1%, 83.4%]
	Sensitivity	92.9%	[87.2%, 98.6%]
	Specificity	29.2%	[19.2%, 39.1%]
Google Gemini	Accuracy	73.8%	[64.1%, 83.4%]
	Sensitivity	87.5%	[80.1%, 94.9%]
	Specificity	41.7%	[32.0%, 51.3%]
Microsoft Copilot	Accuracy	87.5%	[80.4%, 94.6%]
	Sensitivity	94.6%	[90.2%, 99.1%]
	Specificity	70.8%	[59.7%, 82.0%]
DeepSeek AI	Accuracy	68.8%	[58.6%, 78.9%]
	Sensitivity	85.7%	[77.8%, 93.6%]
	Specificity	29.2%	[19.2%, 39.1%]

**Fig 1:**
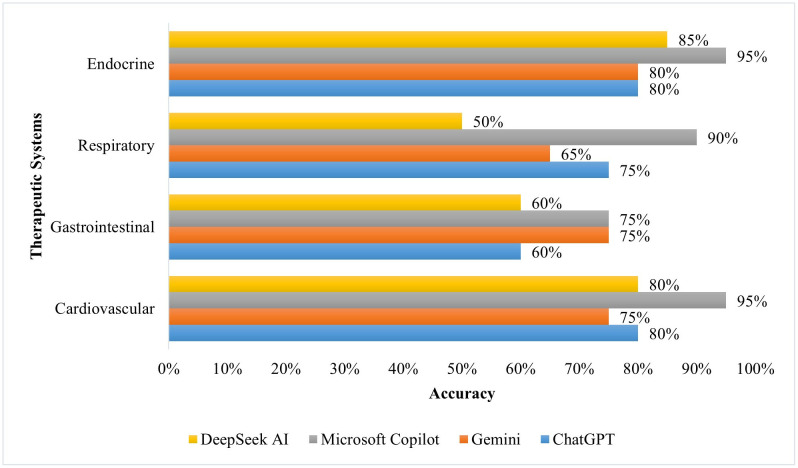
Comparison of AI tool accuracy across therapeutic systems.

**Fig 2:**
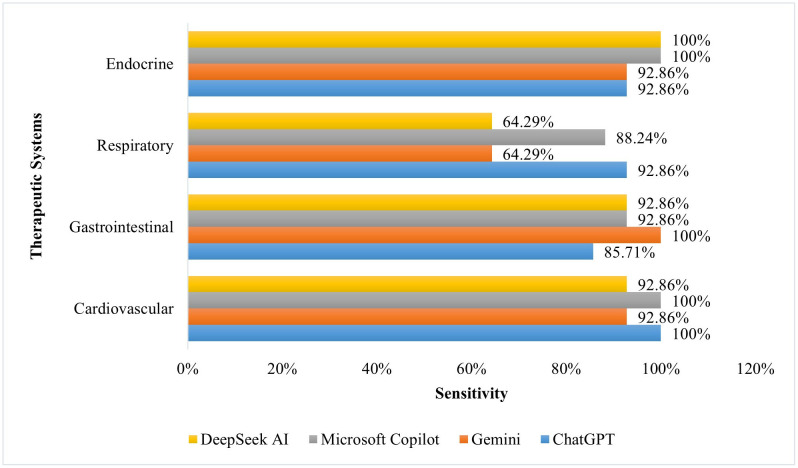
Comparison of AI tool sensitivity across therapeutic systems.

**Fig 3:**
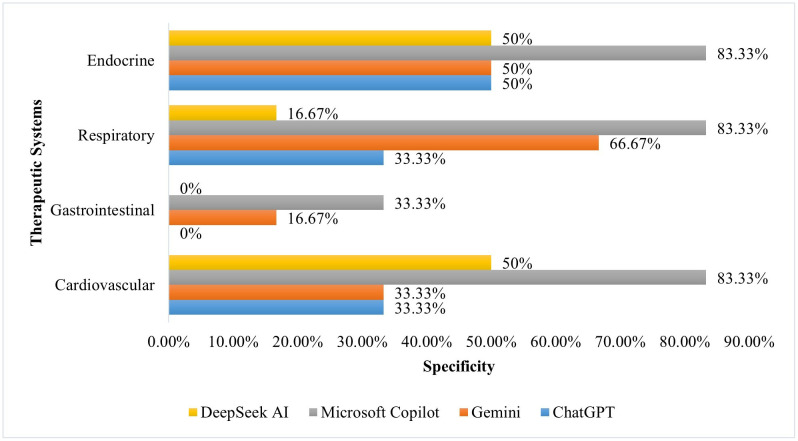
Comparison of AI tool specificity across therapeutic systems.

Figs 1–3 compare the performance of AI tools across the four therapeutic systems. In the Cardiovascular system, Microsoft Copilot obtained the highest accuracy, 95.0%, with perfect sensitivity, 100.0%, and strong specificity, 83.3%. The sensitivity for ChatGPT-3.5 and Google Gemini was also high, 100.0%, but the specificity was low, 33.3%. In the Gastrointestinal system, both Google Gemini and Microsoft Copilot obtained a high accuracy, 75.0%. Microsoft Copilot had high sensitivity, 92.9%, but with poor specificity, 33.3%, whereas the sensitivity of Google Gemini was perfect, 100.0%, while the specificity was low, 16.6%. In the Respiratory system, Microsoft Copilot was also in the lead with an accuracy of 90.0%, supported by high specificity, 83.3%. Lastly, in the Endocrine system, Microsoft Copilot and DeepSeek AI showed a high accuracy, 95.0% and 85.0%, respectively, but with perfect sensitivity, 100.0%, and variable specificity, 83.3% for Microsoft Copilot and 50.0% for DeepSeek AI.

In terms of the performance of the AI in the pharmacological domains (**Table 3**), Microsoft Copilot had the highest accuracy and sensitivity in each domain examined. Within the Mechanism of Action domain, it reached 100.0% for all three metrics. ChatGPT-3.5, Google Gemini, and DeepSeek AI demonstrated an accuracy of 90.0%, but with perfect sensitivity; however, the specificity from these tools was only 50.0%. Within the Side Effects domain, Microsoft Copilot again had the best performance, reaching an accuracy of 95.0% and 100.0% sensitivity, although DeepSeek AI had the lowest performance, which was 60.0% accuracy and 28.6% specificity. In the case of Pharmacokinetics, the tools Google Gemini and Microsoft Copilot both obtained an accuracy of 80.0%, ChatGPT-3.5 had 70.0%, but the sensitivity and specificity did change across the tools. Finally, the Drug Interactions domain showed that Microsoft Copilot led with an accuracy of 75.0% and 85.7% sensitivity, although the rest of the tools showed relatively low specificity at 16.7%.

**Table 3:** Performance of AI tools across pharmacological domain.

Domain	AI Tool	True Positives (TP)	False Negatives (FN)	True Negatives (TN)	False Positives (FP)	Accuracy (%)	Sensitivity (%)	Specificity (%)
Mechanism of Action	ChatGPT-3.5	16	0	2	2	90.0	100.0	50.0
	Google Gemini	16	0	2	2	90.0	100.0	50.0
	Microsoft Copilot	16	0	4	0	100.0	100.0	100.0
	DeepSeek AI	16	0	2	2	90.0	100.0	50.0
Side Effects	ChatGPT-3.5	12	1	3	4	75.0	92.3	42.9
	Google Gemini	11	2	4	3	75.0	84.6	57.1
	Microsoft Copilot	13	0	6	1	95.0	100.0	85.7
	DeepSeek AI	10	3	2	5	60.0	76.9	28.6
Pharmacokinetics	ChatGPT-3.5	13	0	1	6	70.0	100.0	14.3
	Google Gemini	13	1	3	4	80.0	92.9	42.9
	Microsoft Copilot	12	1	4	3	80.0	92.3	57.1
	DeepSeek AI	12	1	2	5	70.0	92.3	28.6
Drug Interactions	ChatGPT-3.5	11	3	1	5	60.0	78.6	16.7
	Google Gemini	10	4	1	5	55.0	71.4	16.7
	Microsoft Copilot	12	2	3	3	75.0	85.7	50.0
	DeepSeek AI	10	4	1	5	55.0	71.4	16.7

All the tools performed very well on easy questions (**Table 4**), where ChatGPT-3.5, Google Gemini, Microsoft Copilot, and DeepSeek AI scored above 92.0%, and both Microsoft Copilot and DeepSeek attained 100.0%. Accuracy declined for average and especially difficult questions, where the proportion of correct responses ranged from 16.7% to 50.0%. Regarding the variation in accuracy across different question difficulties, the difference was statistically significant across all four tools (p < 0.05).

**Table 4:** Comparative performance of AI tools across difficulty levels.

AI Tool	Answer Type	Easy (DI > 0.8)	Average (DI > 0.4–0.8)	Difficult (DI 0–0.4)	p-value^#^
ChatGPT-3.5	Correct	25 (92.6%)	32 (68.1%)	2 (33.3%)	0.003*
	Incorrect	2 (7.4%)	15 (31.9%)	4 (66.7%)	
Google Gemini	Correct	26 (96.3%)	32 (68.1%)	1 (16.7%)	<0.001*
	Incorrect	1 (3.7%)	15 (31.9%)	5 (83.3%)	
Microsoft Copilot	Correct	27 (100.0%)	40 (85.1%)	3 (50.0%)	0.002*
	Incorrect	0 (0.0%)	7 (14.9%)	3 (50.0%)	
DeepSeek AI	Correct	27 (100.0%)	27 (57.4)	1 (16.7%)	<0.001*
	Incorrect	0 (0.0%)	20 (42.6)	5 (83.3%)	

# Using Chi-square test.

* Significant at 0.05 significance level.

The reproducibility of the AI tools was evaluated after two weeks (**Table 5**). The highest reproducibility was shown by DeepSeek AI, giving identical answers in 97.5% of cases (78 out of 80), followed by Google Gemini in 93.8% (75 out of 80), Microsoft Copilot in 91.3% (73 out of 80), and ChatGPT-3.5 in 83.8% (67 out of 80). All tools showed statistical significance in reproducibility (Cohen’s Kappa ranged from 0.621 to 0.942 (p < 0.001)).

**Table 5:** Reproducibility between rounds.

AI Tool	Identical Answers (N)	Total Questions	Reproducibility Index (%)	Cohen’s Kappa	p-value
ChatGPT-3.5	67	80	83.8	0.621	<0.001
Google Gemini	75	80	93.8	0.841	<0.001
Microsoft Copilot	73	80	91.3	0.671	<0.001
DeepSeek AI	78	80	97.5	0.942	<0.001

The performances of the four AI tools were assessed in two test runs, as shown in **Table 6**. ChatGPT and Gemini had slight reductions from 73.8% to 65.0% and 72.5%, respectively. Microsoft Copilot also fell from 87.5% to 81.3%, while DeepSeek AI remained at 68.8%. There were no statistically significant changes for any tool, as detected by the McNemar test with all p-values ≥ 0.05.

**Table 6:** Evaluating accuracy of generative AI tools in multiple test runs.

Tool	Correct (1st Run)	Accuracy (1st Run)	Correct (2nd Run)	Accuracy (2nd Run)	Correct → Incorrect	Incorrect → Correct	*p*-value^#^
ChatGPT-3.5	59	73.8%	52	65.0%	10	3	1.000
Google Gemini	59	73.8%	58	72.5%	3	2	1.000
Microsoft Copilot	70	87.5%	65	81.3%	6	1	0.092
DeepSeek AI	55	68.8%	55	68.8%	1	1	0.125

# Using McNemar test.

## Discussion

As tools like ChatGPT-3.5, Microsoft Copilot, Google Gemini, and DeepSeek become more commonplace in health education, and in pharmacy education in particular, it is important to understand both the strengths and weaknesses of these tools. In this study, 80 questions on pharmacology in the form of MCQs were evaluated using these different AI tools, addressing four therapeutic systems: the cardiovascular system, the gastrointestinal system, the respiratory system, and the endocrine system. Knowledge areas examined were mechanisms of action, side effects, pharmacokinetics, and drug-drug interactions.

Microsoft Copilot reported similar success on the cardiovascular pharmacology tasks, as seen in the work of Salman et al. (2025) [13]; it topped with an overall accuracy of 87.5%, an excellent sensitivity of 94.6%, and good specificity of 70.8%. Since the intervention was likely improved by real-time information available through GPT-4 and Microsoft’s web search capabilities, it probably pulled information from reliable clinical databases such as Drugs.com and Medicine.com [14]. Accuracy, however, still depends on the credibility of its sources. This performance may reflect not only improved access to external databases but also the cognitive scaffolding advantage of retrieval-augmented models, which allows them to overcome some inherent reasoning limitations seen in standalone LLMs [15].

In contrast, DeepSeek AI had the lowest accuracy and specificity: 68.8% and 29.2%, respectively, though its sensitivity was relatively high at 85.7%. Since DeepSeek operates without internet access and cannot update post-deployment, its responses are limited to pretraining data [16]. This possibly explains why it has low performance for current clinical knowledge and may demonstrate the cognitive limitations of static, non-retrieval LLMs, whose performance declines when confronted with updated or nuanced medical data beyond their pretraining corpus [15].

ChatGPT-3.5 and Google Gemini performed with comparable accuracy; otherwise, they differed on other metrics. ChatGPT-3.5 had higher sensitivity, whereas Gemini showed higher specificity. This is also consistent with prior work, including studies by Roos et al. (2023) and Rossettini et al. (2024), which observed similar performance differences between these tools when medical licensure questions were assessed throughout Europe [17,18]. Performance of ChatGPT may be favored by reinforcement learning with human feedback [13], whereas Gemini has not been optimized for highly specialized content such as pharmacology [19].

Microsoft Copilot had the highest performance of the four therapeutic systems, demonstrating perfect sensitivity and high specificity for cardiovascular topics, as well as high accuracy in the respiratory system [20]. This reflects a strong ability to discriminate between correct and incorrect responses. A prior study utilizing ChatGPT-4.0 demonstrated its superior performance to Copilot (then Bing), but this likely reflects our use of the less mature ChatGPT-3.5 system compared to that used in ChatGPT-4.0 [20]. Tools such as Copilot that are capable of real-time access to web data tend to generate higher performances compared to those tools that are confined to static training sets, such as DeepSeek AI. Real-time retrieval may reduce bias propagation by incorporating current, externally verified content, unlike static models that tend to reinforce training-set biases [21]. All tools did well in answering mechanism-of-action questions, likely due to the consistency of that domain across datasets. However, more variability appeared across pharmacokinetics and drug interactions, thus reinforcing a continued need for validation of AI clinical education tools [14].

When stratified by level of difficulty, all AI tools performed well on easy questions, which suggests a good capability for fundamental knowledge. However, when increasing the difficulty, performance fell. This decline likely reflects the limited higher-order reasoning capacity of current LLMs [22]. Microsoft Copilot correctly answered 100.0% of easy questions and 85.0% of the moderately difficult questions, which maintains the best overall consistency. In contrast, the accuracy of Gemini decreased to 16.7% with difficult questions and ChatGPT-3.5 performed a little better at 33.3%. The results are reflected in earlier studies, whereby current AI performs poorly on higher-order reasoning and subtle clinical decisions [13,23,24].

In general, a substantial difference in performance according to difficulty was established, with p < 0.05, suggesting that question difficulty remains an important consideration for use with AI-based learning. Microsoft Copilot seems ready to be included in educational learning, but there are mixed results with other tools, particularly with more advanced questions that could warrant caution with its use. Significantly, ChatGPT-4.0 was found to perform better than Microsoft Copilot on advanced medical questions in previous studies [20,25], which indicates the importance of verifying the version of the tool being used. In this study, we used ChatGPT-3.5, which is a free version and, therefore, offers less advanced tool functionality than GPT-4, which could have influenced our results.

Another important dimension of assessment was reproducibility. DeepSeek AI provided the highest degree of reproducibility over multiple runs, at 97.5%, whereas ChatGPT-3.5 was the least stable-it sometimes gave different answers to the same question. This variability underscores a known limitation in LLMs: stochastic generation and sensitivity to prompt and session context, which can cause shifts in answer formulation despite identical inputs [26]. This was in agreement with the recent works of Gao et al. (2023) and Giannakopoulos et al. (2023), who concluded that the responses of Large Language Models (LLMs) are variable in time and context [14,27] Even though high reproducibility is often viewed as an opportunity, as has been seen, there can be a lethal paradox when low accuracy is paired with high reproducibility. In educational contexts, this would be problematic. For example, high reproducibility would indicate that DeepSeek AI would return the same response each time, but if that response was incorrect, any student or learner would reinforce faulty information. This could be particularly dangerous for the academic or clinical settings, where accuracy and precision are typically the focus.

Despite their limitations, AI tools are evolving rapidly and demonstrate a very clear value for supporting learning, particularly of basic and intermediate content. Yet they should be used with, not instead of, human educators where clinical application and decision-making occur.

Limitations of this study included that it assessed only freely available versions of each AI platform; paid or updated versions, such as ChatGPT-5, may yield much higher performances, especially on complex questions. However, considering that a large number of users will use free tools, this approach reflects common real-world usage. In addition, the framing and phrasing of questions can significantly affect the output of AI, which may indicate a need for standardized prompt design in future research. Moreover, the relatively small sample of 80 MCQs may not fully reflect the broad range and diversity of pharmacology topics, which could limit the generalizability of the findings. Also, the research was conducted entirely in the English language, focusing on the performance of pharmacology questions, which may be different from multilinguistic or other health domains. Finally, AI is ever-evolving, and the results may be outdated with the release of newer tools and features; thus, there is a need to continuously monitor and evaluate the performance using newer systems.

## Conclusion

This investigation compared the performance of four AI tools, Microsoft Copilot, ChatGPT-3.5, Google Gemini, and DeepSeek AI, on 80 pharmacology-related MCQs. Microsoft Copilot had the best overall accuracy, sensitivity, and specificity, while DeepSeek had the best reproducibility despite its lower accuracy. All the tested tools had good performance for simple questions, but their performance decreased with increasing complexity. Performances are not always significantly different, and inconsistencies, particularly of ChatGPT-3.5, make caution necessary.

AI tools may serve as a supplement to pharmacology education, but their content must be verified with standard resources in pharmacology education (or with a trusted source) for accuracy. Instructors should utilize AI tools to help reinforce basic knowledge and support easier ideas; however, they should not serve as a replacement for standard study methods, especially for more complicated clinical scenarios. Pharmacology students should also be encouraged to cross-check AI responses with a standard resource and seek assistance from their instructors for more difficult subject content. Ultimately, AI tools, based on their limitations, should be utilized as a supplement to standard learning until these capabilities or limitations improve.

## Supporting information

S1 Appendix Study MCQs on pharmacology. (DOCX)

S2 Appendix Raw data. (XLSX)
