# Supplementary material for: Diagnostic performance of four AI tools in pharmacology MCQs: Accuracy, sensitivity, and specificity
Source: PLoS One. 2025 Dec 16;20(12):e0337688. doi: 10.1371/journal.pone.0337688 (PMC12707663; doi:10.1371/journal.pone.0337688)
Supplement: S1 Appendix — (DOCX) [file pone.0337688.s001.docx]

**The Multiple Choice Questions**

| **Systems** | **Categories** | **Questions 80** |
| --- | --- | --- |
| Cardiovascular | Mechanism of action | 1. **What is the primary mechanism of action of nitrates in the treatment of angina?** a) Vasodilation of coronary arteries b) Inhibition of platelet aggregation c) Increase in myocardial oxygen demand d) Decrease in blood glucose levels   Answer: a) Vasodilation of coronary arteries   1. **What is the primary mechanism by which fluvastatin lowers cholesterol levels?** a) Inhibition of bile acid absorption b) Activation of lipoprotein lipase c) Competitive inhibition of HMG-CoA reductase d) Non-competitive inhibition of HMG-CoA reductase   Answer: c) Competitive inhibition of HMG-CoA reductase   1. **What is the mechanism by which clopidogrel works?** 2. Inhibition of thromboxane A2 3. Irreversible binding to P2Y12 ADP receptors 4. Activation of GPIIb/IIIa complex 5. Reversible binding to P2Y12 ADP receptors   Answer b) Irreversible binding to P2Y12 ADP receptors   1. **How does Rivaroxaban work in the body?** 2. Direct, selective, and reversible inhibition of thrombin (factor IIa) in both the intrinsic and extrinsic coagulation pathways. 3. Direct, selective, and reversible inhibition of factor Xa in intrinsic and extrinsic coagulation pathways. 4. Activation of antithrombin III. 5. Inhibition of platelet aggregation.   Answer: b) direct, selective, and reversible inhibition of factor Xa in intrinsic and extrinsic coagulation pathways.   1. **How does Tolvaptan initially work in the body?** 2. Vasopressin V1 receptor agonist. 3. Vasopressin V2 and V1a in a ratio of 29:29 receptor antagonist 4. Vasopressin V2 and V1a in a ratio of 29:25 antagonist 5. Sodium channel blocker   Answer: None (Vasopressin V2 and V1a in a ratio of 29:1 receptor antagonist). |
| Cardiovascular | Side effects | 1. **What side effect is commonly associated with statins?**   a) Arrhythmias  b) Myalgia  c) Peripheral neuropathy  d) Hyperkalemia  Answer: b) Myalgia   1. **What is the common side effect of Dihydropyridine calcium channel blockers?**   a) Bradycardia  b) Peripheral edema  c) Increased urination  d) Hypoglycemia  Answer: b) Peripheral edema   1. **What health complication can arise from amiodarone toxicity?**   a) Liver failure  b) Pulmonary fibrosis  c) Renal failure  d) Anemia  Answer: b) Pulmonary fibrosis   1. **Which medication can lead to cyanide poisoning?**   a) Nitroglycerin  b) Nitroprusside  c) Verapamil  d) Hydralazine  Answer: b) Nitroprusside   1. **What is a common side effect of taking Candesartan?** 2. Septic arithritis 3. Hypokalemia 4. Fever 5. Hypoglycemia   Answer: None (Hyperkalemia) |
| Cardiovascular | Drug-drug interactions | 1. **What risk should be considered when combining Acipimox with statins like Rosuvastatin?** 2. Increased risk of hypertension 3. Enhanced myopathic effect (rhabdomyolysis) 4. Decreased cholesterol levels 5. Increased Cystitis   Answer: b) Enhanced myopathic effect (rhabdomyolysis)   1. **What is the potential risk or side effect of taking verapamil and beta-blockers together?** 2. Hypertension 3. Severe tachycardia 4. Hyperkalemia 5. Diarrhea   Answer: None (Severe bradycardia)   1. **Which medication is known to enhance the anticoagulant effect of warfarin?** 2. Rifampin 3. Edrophonium 4. Phaenobarbital 5. Carbamazepine   Answer: None (Amiodarone)   1. **Amiodarone increase the toxicity of anticonvulsants which drug is most likely affected?** 2. Carbamazepine 3. Phenytoin 4. Valproate 5. Cannabidiol   Answer: b) Phenytoin.   1. **Which drug is known to enhance the blood pressure-lowering effects of aprocitentan?** 2. Acetylsalicylic acid 3. Amlodipine 4. Ibuprofen 5. Cefixim   Answer: b) Amlodipine |
| Cardiovascular | Pharmacokinetics | 1. **How quickly does amiodarone begin to take effect when taken orally?** 2. 12 hours 3. 14 days to 12 weeks 4. 2 days to 3 weeks 5. 7 days   Answer: c) 2 days to 3 weeks   1. **How long does warfarin stay effective in the body?** 2. 9 days to 10 days 3. 5 to 6 hours 4. 1 to 2 hours 5. 12 to 24 hours   Answer: None (2-5days)   1. **What is the half-life of digoxin in adults?** 2. 2 to 4 hours 3. 8 to 12 hours 4. 24 to 36 hours 5. 36 to 48 hours   Answer: d) 36 to 48 hours   1. **What percentage of diltiazem binds to plasma proteins?** 2. 100% 3. 10 to 20% 4. 85% to 90% 5. 50 to 60%   Answer: None (70 to 80)   1. **How often is inclisiran given after the first dose?** 2. After 2 months then every 12 months 3. After 3 months then every 6 months 4. After 6 months then every 8 months 5. Every 12 months   Answer: b) After 3 months then every 6 months |
| Gastrointestinal tract | Mechanism of action | 1. **What is the mechanism by which metoclopramide works?** 2. Inhibition of gastric acid secretion 3. Stimulation of gastric motility and antiemetic action 4. Binding to opioid receptors in the GI tract 5. Decreasing the rate of gastric emptying   Answer: b) Stimulation of gastric motility and antiemetic action   1. **How does zolbetuximab-clzb work to treat stomach cancer?** 2. It inhibits DNA synthesis in tumor cells. 3. It targets CLDN18.2-positive cells and induces cell death via ADCC and CDC. 4. It blocks VEGF signaling to inhibit angiogenesis. 5. It acts as a kinase inhibitor for CLDN18.2-positive cells.   Answer: b) It targets CLDN18.2-positive cells and induces cell death via ADCC and CDC.   1. **How does ranitidine work to reduce stomach acid?** 2. Inhibiting proton pumps 3. Blocking H2 histamine receptors 4. Stimulating gastric motility 5. Increasing mucus production   Answer: b) Blocking H2 histamine receptors.   1. **How does bismuth subsalicylate work in the body?** 2. Inhibiting gastric acid secretion 3. Neutralizing gastric acid 4. Anti-secretory and antimicrobial 5. Stimulating gastric motility   Answer: c) Anti-secretory and antimicrobial   1. **How does secnidazole primary work to treat infections?**   a) Inhibiting protein synthesis  b) Interfering with bacterial mitochondria  c) Blocking cell wall synthesis  d) Neutralizing gastric acid  Answer: None (Interfering with bacterial DNA synthesis) |
| Gastrointestinal tract | Side effects | 1. **What is the common side effect of H2 receptor antagonists, such as ranitidine?** 2. Dry mouth 3. Drowsiness 4. Diarrhea 5. Liver toxicity   Answer: b) Drowsiness   1. **What side effect can occur with long-term use of misoprostol?** 2. Constipation 3. Diarrhea 4. Hyperkalemia 5. Tachycardia   Answer: b) Diarrhea   1. **Which type of antacid is most likely to lead to increased urination?** 2. Magnesium hydroxide 3. Aluminum hydroxide 4. Calcium carbonate 5. Potassium hydroxide   Answer: None (Sodium bicarbonate)   1. **What deficiency can occur with long-term use of rabeprazole?** 2. Vitamin C 3. Vitamin A 4. Vitamin K 5. Vitamin E   Answer: None (Vitamin B12)   1. **What condition can develop in men with prolonged use of cimetidine?** 2. Gynecomastia 3. Hyperglycemia 4. Hypotension 5. Anemia   Answer: a) Gynecomastia. |
| Gastrointestinal tract | Drug-drug interactions | 1. **Why is it recommended to avoid taking antacids with tetracycline?** 2. Antacids reduce the absorption of tetracyclines 3. Antacids increase the absorption of tetracyclines 4. Tetracyclines neutralize the effect of antacids 5. No interaction occurs   Answer: a) Antacids reduce the absorption of tetracyclines   1. **What happens when aluminum-containing antacid is taken at the same time as digoxin?**   a) Increased bioavailability of digoxin  b) Decreased absorption of digoxin  c) No interaction  d) Enhanced renal excretion of digoxin  Answer: b) Decreased absorption of digoxin   1. **Which drug has reduced metabolism when taken with cimetidine?** 2. Warfarin 3. Acetaminophen 4. Ibuprofen 5. Aspirin   Answer: a) Warfarin.   1. **Which medication has reduced absorption when taken with aluminum hydroxide?** 2. Levofloxacin 3. Ibuprofen 4. Metformin 5. Simvastatin   Answer: a) Levofloxacin.   1. **Which drug becomes less effective when taken with rabeprazole?** 2. Paracetamol 3. Ibuprofen 4. Acetaminophen 5. Amoxicillin   Answer: None (Clopidogrel). |
| Gastrointestinal tract | Pharmacokinetics | 1. **How quickly does pantoprazole start working when taken orally or intravenously?** 2. Oral 2.5 hours and IV 15 to 30 minutes 3. Oral 5 hours and IV 15 to 30 minutes 4. Oral 2.5 hours and IV 1 to 2 hours 5. Oral 8 hours and IV 30 to 40 minutes   Answer: a) Oral 2.5 hours and IV 15 to 30 minutes   1. **Which proton pump inhibitor (PPI) has the highest bioavailability when taken orally?** 2. Omeprazole 3. Lansoprazole 4. Pantoprazole 5. Rabeprazole   Answer: b) Lansoprazole.   1. **What is the usual time it takes for loperamide to be eliminated from the body?**   a) 5.5 to 7.1 hours  b) 18 to 24 hours  c) 36 to 48 hours  d) 48 to 58 hours  Answer: None (9.1 to 14.4 hours).   1. **What percentage of metronidazole is excreted unchanged in the urine?** 2. Less than 5% 3. Approximately 10% 4. About 70% 5. More than 50%   Answer: None (About 20%).   1. **How long does it usually take for sucralfate to start working after being taken orally?** a) 15 to 30 minutes b) 1 to 2 hours c) 3 to 4 hours d) 6 to 8 hours   Answer: b) 1 to 2 hours. |
| Respiratory | Mechanism of action | 1. **How does Salbutamol (albuterol) primarily work in the body?** 2. Relaxes bronchial smooth muscle by action on beta2 receptors. 3. Relaxes bronchial smooth muscle by action on beta2 and beta1 receptors. 4. Relaxes bronchial smooth muscle by action on alpha1 receptors. 5. contraction of bronchial smooth muscle by action on beta2 receptors.   Answer: a) Relaxes bronchial smooth muscle by action on beta2 receptors   1. **How does Montelukast work in the body?** 2. Selective leukotriene receptor agonist 3. Selective leukotriene receptor antagonist 4. Non-selective leukotriene receptor antagonist 5. Non-selective leukotriene receptor antagonist   Answer: b) Selective leukotriene receptor antagonist   1. **How does Formoterol work in the body?** 2. Long-acting effect on alpha1 receptors 3. short-acting effect on beta2 receptors 4. short-acting effect on beta1 receptors 5. short-acting effect on beta2 and alpha1 receptors   Answer: None (long-acting effect on beta2 receptors.)   1. **How does the corticosteroid fluticasone work in the body?** 2. Vasodilation and anti-inflammatory activity 3. Inhibit Beta-1 and anti-inflammatory activity. 4. Inhibit alpha-1 and anti-inflammatory activity. 5. Vasoconstrictive and anti-inflammatory activity   Answer: d) Vasoconstrictive and anti-inflammatory activity   1. **What does cetirizine do in the body?** 2. Competes with histamine for H2 receptor 3. Competes with histamine for H1 receptor 4. Competes with histamine for H1 and H2 receptor 5. Competes with histamine for Beta2 receptor   Answer: b) Competes with histamine for H1 receptor |
| Respiratory | Side effects | 1. **What is the common side effect that people often experience when taking Salbutamol (albuterol)?** 2. Hypertension 3. Diabetes mellitus 4. Tinnitus 5. Pharyngitis   Answer: d) Pharyngitis   1. **What level in the body is typically increased when taking Montelukast?** 2. Increased serum B12 3. Increased vitamin A 4. Increased serum AST 5. Increased Mg   Answer: c) Increased serum AST   1. **What is the U.S. boxed warning associated with the use of Formoterol?** 2. Asthma-related deaths 3. Hypertension 4. Diabetes-related deaths 5. Tachycardia   Answer: a) Asthma-related deaths   1. **What is the common side effect of taking theophylline?** 2. Hypocalcemia 3. Hypermagnesemia 4. Hypophosphatemia 5. Hypernatremia   Answer: None   1. **What is the side effect of taking ipratropium?** 2. Parkinson's disease 3. Severe liver damage 4. Loss weight 5. Leg pain   Answer: None |
| Respiratory | Drug-drug interactions | 1. **What can enhance the tachycardia effect of the beta2-agonist terbutaline?** 2. Atomoxetine 3. Enalapril 4. Metformin 5. Paracetamol   Answer: a) Atomoxetine   1. **What drug, when taken with salmeterol, might increase the risk of pulmonary edema and/or dyspnea?** 2. Warfarin 3. Rabeprazole 4. celecoxib 5. Paracetamol   Answer: None (Atosiban)   1. **What happens when fluticasone oral inhaler is taken with desmopressin?** 2. Enhance hypernatremic effect 3. Enhance hypocalcemic effect 4. Enhance hypokalemic effect 5. Enhance hyperkalemic effect   Answer: None (Enhance hyponatremic effect)   1. **What drug can lower the serum concentration of theophylline derivatives?** 2. Alcohol 3. Allopurinol 4. Adalimumab 5. Antithyroid Agents   Answer: c) Adalimumab   1. **What can increase the anticholinergic effect of ipratropium?** 2. Aclidinium 3. Acetylcholinesterase Inhibitors 4. Valsartan 5. Captopril   Answer: a) Aclidinium |
| Respiratory | Pharmacokinetics | 1. **What percentage of betamethasone is bound to proteins in the body?** 2. 64% 3. 90% 4. 100% 5. 40%   Answer: a) 64%   1. **What is the Half-life elimination of Montelukast?** 2. 6.5 to 8 hours 3. 2.7 to 5.5 hours 4. 30 minutes 5. 1 hour   Answer: b) 2.7 to 5.5 hours   1. **How quickly does terbutaline start working when given subcutaneously?** 2. 30 minutes 3. 1 hour 4. 6 to 15 minutes 5. 2 to 3 minute   Answer: c) 6 to 15 minute   1. **What is half-life elimination in children of cetirizine?** 2. 6.2 hours 3. 8.2 hours 4. 12.5 hours 5. 4.2 hours   Answer: a) 6.2 hours   1. **What percentage of theophylline is excreted unchanged in the urine of neonates?** 2. ~10% 3. ~80% 4. ~20% 5. ~100%   Answer: None (~50%) |
| Endocrine | Mechanism of action | 1. **How does sitagliptin primarily work in the body?** 2. SGLT2 inhibitors 3. GLP1 receptor agonists 4. Dipeptidyl Peptidase 4 (DPP-4) Inhibitor 5. Sulfonylureas   Answer: c) Dipeptidyl Peptidase 4 (DPP-4) Inhibitor   1. **How does methimazole work?** 2. Inhibits the synthesis of thyroid hormones by blocking the oxidation of iodine and blocking synthesis of thyroxine and triiodothyronine (T3); does not inactivate circulating T4 and T3. 3. Inhibits the synthesis of thyroid hormones by blocking the oxidation of iodine and blocks the synthesis of thyroxine and triiodothyronine (T3); also inactivate circulating T4 and T3. 4. Enhance the synthesis of thyroid hormones by oxidation of iodine and synthesis of thyroxine and triiodothyronine (T3). 5. Enhance the synthesis of thyroid hormones by oxidation of iodine and synthesis of thyroxine only.   Answer: a) Inhibits the synthesis of thyroid hormones by blocking the oxidation of iodine and blocking the synthesis of thyroxine and triiodothyronine (T3); does not inactivate circulating T4 and T3.   1. **What is the main way that cabergoline works in the body?** 2. Short-acting dopamine receptor agonist with a high affinity for D2. 3. Long-acting dopamine receptor agonist with a high affinity for both D2 and D1. 4. Long-acting dopamine receptor agonist with a high affinity for D2. 5. Short-acting dopamine receptor agonist with a high affinity for both D2 and D1.   Answer: c) Long-acting dopamine receptor agonist with a high affinity for D2.   1. **What is the correct description of how repaglinide works in the body?** 2. Blocks Nicotinic Acetylcholine Receptors 3. Voltage-Gated Calcium Channels 4. Blocks ATP-dependent potassium channels 5. Voltage-Gated Sodium Channels   Answer: c) Blocks ATP-dependent potassium channels   1. **What is the main mechanism of action of Pioglitazone?** 2. Potent and selective antagonist for peroxisome proliferator-activated receptor-gamma (PPAR gamma) 3. Potent and Non-selective antagonist for peroxisome proliferator-activated receptor-gamma (PPAR gamma) 4. Potent and selective GLP-1 receptor agonists 5. Potent and selective GLP-1 receptor antagonists   Answer: None |
| Endocrine 20 | Side effects | 1. **What is side effect happen when take Sitagliptin?** 2. Nasopharyngitis 3. Constipation 4. Decreased creatinine 5. Vomiting   Answer: a) Nasopharyngitis   1. **What level in the body may increase when taking levothyroxine?** 2. Increased B12 3. Increased creatinine 4. Increased WBC 5. Increased liver enzymes   Answer: d) Increased liver enzymes   1. **What is side effect may happen when take pioglitazone?** 2. Increased serum triglycerides 3. Decreased HDL-cholesterol 4. Increased hematocrit 5. Increased hemoglobin   Answer: None   1. **What is a correct side effect associated with metformin?** 2. Constipation 3. Flatulence 4. Hypertension 5. Bradycardia   Answer: b) Flatulence   1. **What is the correct side effect associated with Glipizide?** 2. Decreased lactate dehydrogenase 3. Decreased serum alkaline phosphatase 4. Decreased serum AST 5. Decreased serum creatinine   Answer: None |
| Endocrine | Drug-drug interactions | 1. **What drug can reduce the effectiveness of thyroid products like levothyroxine?** 2. Piracetam 3. Diltiazim 4. Warfarin 5. Apalutamide   Answer: d) Apalutamide   1. **What drug can enhance the hypoglycemic effect of repaglinide, an antidiabetic agent?** 2. Hyperglycemia-Associated Agents 3. Alpha-Lipoic Acid 4. Ritodrine 5. Thiazide and Thiazide-Like Diuretics   Answer: b) Alpha-Lipoic Acid   1. **What drug can decrease the serum concentration of MetFORMIN?** 2. Bupropion 3. Bictegravir 4. Abemaciclib 5. Cephalexin   Answer: None   1. **What drug may decrease the hypoglycemic effect of Sulfonylureas (Glipizide)?** 2. Fibric Acid Derivatives 3. Cyclic Antidepressants 4. Beta-Blockers 5. Antidiabetic Agents   Answer: None   1. **What drug can increase the serum concentration of Rosiglitazone?** 2. Letermovir 3. Paracetamol 4. Ibuprofen 5. Enalapril   Answer: a) Letermovir |
| Endocrine | Pharmacokinetics | 1. **What is the bioavailability of methimazole?** 2. 100% 3. 93% 4. 50% 5. 20%   Answer: b) 93%   1. **What is the initial dose of pioglitazone in adults?** 2. 15 to 30 mg once daily 3. 15 to 35 mg once daily 4. 15 to 40 mg once daily 5. 40 to 60 mg once daily   Answer: a) 15 to 30 mg once daily   1. **What is the primary excretion of Carbergolin?** 2. Urine 60% 3. Feces 90% 4. Feces 60% 5. Urine 80%   Answer: c) Feces 60%   1. **What dose of metformin should be used if the estimated glomerular filtration rate (eGFR) is less than 30 mL/minute/1.73 m²?** 2. Give metformin 850 twice a day 3. Use is contraindicated 4. Give 500 twice a day 5. Give 500 once a day   Answer: b) Use is contraindicated   1. **What is the recommended dose of metformin for patients with end-stage renal disease (ESRD)?** 2. Initial dose of 10 mg once daily with titration as needed has been recommended 3. Initial dose of 5 mg once daily with titration as needed has been recommended 4. Initial dose of 5 to 10 mg once daily with titration as needed has been recommended 5. Initial dose of 10 to 20 mg once daily with titration as needed has been recommended   Answer: None |
